# Targeting Receptors on Cancer Cells with Protein Toxins

**DOI:** 10.3390/biom10091331

**Published:** 2020-09-17

**Authors:** Antonella Antignani, Eric Chun Hei Ho, Maria Teresa Bilotta, Rong Qiu, Robert Sarnvosky, David J. FitzGerald

**Affiliations:** Biotherapy Section, Laboratory of Molecular Biology, Center of Cancer Research, National Cancer Institute, NIH., 37 Convent Dr, Room 5124, Bethesda, MD 20892, USA; ericchunhei.ho@nih.gov (E.C.H.H.); maria.bilotta@nih.gov (M.T.B.); rong.qiu@nih.gov (R.Q.); sarnovsr@pop.nci.nih.gov (R.S.)

**Keywords:** toxin, immunotoxin, diphtheria, pseudomonas, ricin, receptor, cancer

## Abstract

Cancer cells frequently upregulate surface receptors that promote growth and survival. These receptors constitute valid targets for intervention. One strategy involves the delivery of toxic payloads with the goal of killing those cancer cells with high receptor levels. Delivery can be accomplished by attaching a toxic payload to either a receptor-binding antibody or a receptor-binding ligand. Generally, the cell-binding domain of the toxin is replaced with a ligand or antibody that dictates a new binding specificity. The advantage of this “immunotoxin” approach lies in the potency of these chimeric molecules for killing cancer cells. However, receptor expression on normal tissue represents a significant obstacle to therapeutic intervention.

## 1. Introduction

**Figure 1:**
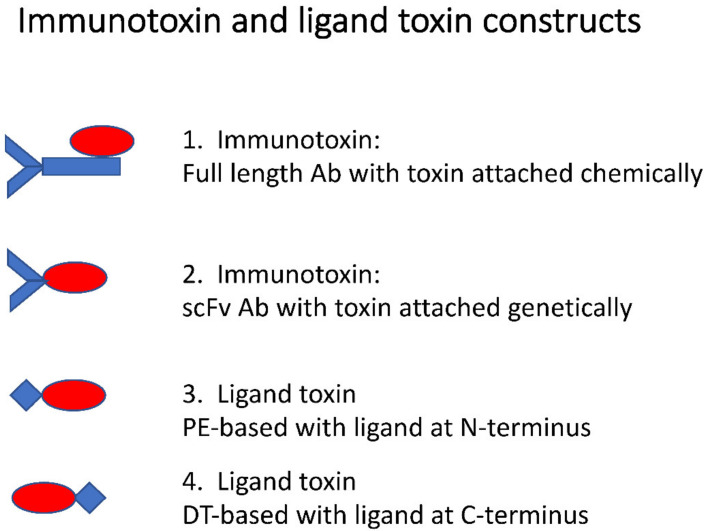
Immunotoxin and ligand toxin constructs. In examples 1 and 2, antibodies (blue) are joined with toxins (red) to form immunotoxins. Shown in example 1, a toxin is attached chemically to a full-length antibody. Example 2 is a genetic fusion between the single-chain Fv portion of an antibody and a toxin. In examples 3 and 4, the two most common ligand toxin constructs are shown. Example 3 shows a ligand toxin whereby the ligand (blue) is placed at the *N*-terminus of the construct, in place of pseudomonas exotoxin’s (PE’s) native binding domain. Example 4 shows the ligand at the *C*-terminus, replacing the native binding domain of diphtheria toxin (DT).

Cancer cells frequently upregulate surface receptors that promote growth and survival. These receptors constitute valid targets for intervention. One strategy involves the delivery of toxic payloads with the goal of killing those cancer cells with high levels of specific receptors. Delivery can be accomplished by attaching a toxic payload to either a receptor-binding antibody or ligand. Antibody-toxin agents are called “immunotoxins” while ligand toxins retain their own designation. Receptors provide an entry pathway for the delivery of cytotoxic drugs or proteins to the cell interior. Here, we confine our discussion to the use of bacterial or plant protein toxins that act catalytically within cells to inhibit protein synthesis. Generally, the cell-binding domain of the toxin is replaced with a ligand or antibody that dictates a new binding specificity (Figure 1). Further, while this review will cover both antibody-toxin and ligand-toxin therapeutics, it is important to highlight key differences among these two types of chimeric molecules. Anti-receptor antibodies rarely stimulate signaling pathways while ligands certainly will. Additionally, the effectiveness of ligand-toxins can be blocked by high local concentrations of endogenous ligand which is rarely an issue with antibody-based agents. Finally, anti-receptor antibodies, unlike ligands, can target portions of the external domains of receptors that are not directly involved in ligand binding—thus there may be many more binding sites available to antibodies than to ligands.

**Figure 2:**
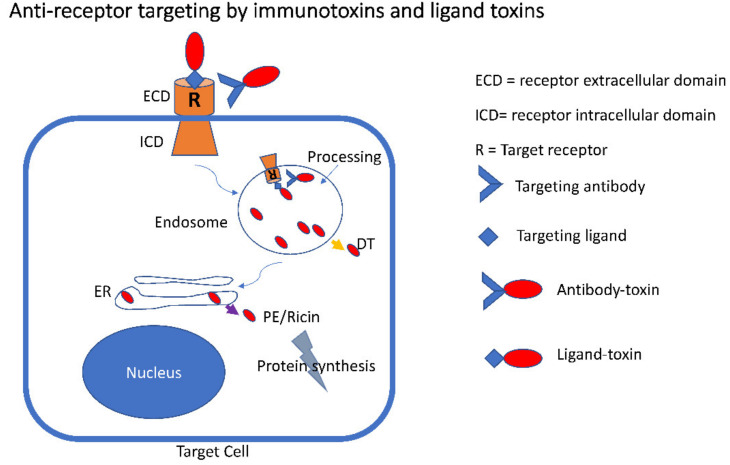
Anti-receptor targeting by immunotoxins and ligand toxins. A ligand toxin is shown interacting with a target receptor at the ligand-binding site. Similarly, an immunotoxin is shown binding the same receptor but at a distinct site. Following binding, internalization results in delivery to endosomes. In endosomes, toxins are processed (often by furin-like proteases) to separate the antibody or ligand from the toxin. After the processing step, some toxins such as DT translocate directly from endosomes (yellow arrow) to the cell cytosol while others traffic further into the cell to the endoplasmic reticulum where translocation is noted for pseudomonas exotoxin (PE) and some plant toxins (purple arrow). Once in the cytosol, toxins shut down protein synthesis.

Receptors, especially those tied to oncogenic progression, represent attractive targets. In addition to high expression levels, surface receptors can provide an efficient gateway for internalization (Figure 2). Normally, internalization by signaling receptors (e.g., growth factor or cytokine receptors) leads to receptor downregulation and destruction of both ligand and receptor. Targeted toxins can use the receptor internalization feature but must avoid destruction. Likewise, internalization by nutrient-related (e.g., lipoprotein or transferrin receptors) receptors allows for cargo uptake but recycling of the receptor to the cell surface allows additional rounds of internalization. For these receptors, the toxin must leave the recycling pathway—or risk being returned to the media. In either case, it is possible to deliver cytotoxic payloads to surface receptors on cancer cells. However, receptor expression on normal tissue represents a significant obstacle to therapeutic intervention.

Below, we focus first on targeting the epidermal growth factor receptor (EGFR) and related receptors, then consider the transferrin receptor (TFR), interleukin receptors and finally a “basket” of other receptors.

## 2. Immunotoxin Production

Protein toxins are lethal for mammalian cells. This has led to production schemes where bacterial expression systems are used to make the entire immunotoxin or the toxin component of the immunotoxin. A standard method for pseudomonas exotoxin (PE)-based immunotoxins has been developed [1]. Plant toxins are usually attached to antibodies using chemical cross-linking agents. Methods for this have been well described [2,3]. It has also been possible to construct cytotoxic immunotoxins using gene fusion technology with gelonin as the toxin [4].

## 3. Immunotoxins Targeting EGFR

Several decades ago, EGFR was identified as an attractive cancer target because a high level of expression led to oncogenesis [5]. The high expression was due in many cases to gene amplification [6]. EGFR mutants are also identified with activating mutations including point mutations and large deletions [7,8]. Structurally, EGFR has a large extracellular domain with four subdomains, a transmembrane domain and an intracellular tyrosine kinase domain [7]. Ligand binding to the extracellular domain leads to receptor dimerization, internalization and receptor degradation [7]. Because of efficient internalization, EGFR was considered an attractive target for the delivery of cytotoxic compounds. While EGFR can bind up to twelve distinct ligands, cancer targeting has been attempted mainly with two ligands, EGF and TGF-alpha. Targeting with anti-receptor antibodies has also been attempted (see below). Historically, ligand-toxins targeting EGFR were produced early on and only after many years were toxic payloads added to receptor-binding antibodies.

Immunotoxins against EGFR were first produced in the 1980s where EGFR ligands were attached chemically or fused with toxins [9,10]. The first attempt at making an EGFR-targeted ligand-based immunotoxin was the fusing TGFα and to a fragment of PE, to produce TGFα-PE40 (TP40) [9]. This immunotoxin was cytotoxic for cell lines that overexpressed EGFR (e.g., epidermoid carcinoma). In vivo studies reported on the enhanced survival of nude mice bearing EGFR expressing xenografts [11]. In a Phase I clinical trial, TP40 was tested as a treatment for patients with bladder cancer [12]. TP40 was well tolerated with no dose-limiting toxicity. Patients with the invasive disease showed no evidence of antitumor activity, whereas eight of nine patients with carcinoma in situ exhibited clinical improvement following therapy [12]. A reengineered TP40, TP38, was made with a slightly smaller truncated PE, that was more potent. TP38 was tested in a Phase I trial of patients with malignant brain tumors using intracerebral infusion. Three of the fifteen patients demonstrated radiographic responses, with one patient exhibiting nearly complete response and remained alive >260 weeks after therapy, two dose-limiting toxicities, grade 3 hemiparesis and grade 4 fatigue, prevented the study from achieving the planned maximum tolerated dose [13,14]. In addition, leakage of the infusion to the surrounding area of the tumor was observed in >80% of the patients [13]. Given the dose-limiting toxicity and infusion leakage, no further trial was pursued.

EGF was also used to construct ligand-based immunotoxins. A fusion of EGF and the catalytic and translocation domains of diphtheria toxin (DT) produced DAB_389_EGF [15]. DAB_389_EGF was cytotoxic for various EGFR-expressing tumor cell lines with IC50s values as low as 0.1 pM [15]. A Phase I/II clinical trial of DAB_389_EGF was conducted in 52 patients with metastatic cancers [16]. There were a few minor responses with all the patients developing anti-DT or anti-EGF neutralizing antibodies. Dose-limiting toxicities were due to liver or kidney damage and chest pain [16].

Another approach for targeting this receptor is to produce immunotoxins using anti-EGFR antibodies. One such effort employed single-chain variable fragments (scFv) derived from the antibodies Cetuximab or Panitumumab [17]. The scFvs were fused to PE and the two immunotoxins derived from either Cetuximab (scFv2112-ETA) or Panitumumab (scFv1711-ETA) were tested against multiple EGFR overexpressing cell lines. Both immunotoxins showed cytotoxicity toward the target cells with IC50 values of 4 pM to 460 pM depending on the cell line. In addition, the immunotoxins showed binding to patient sample tissue. Although preclinical results were promising, no clinical trials were pursued.

## 4. EGFRvIII and “Cancer-Expressed” EGFR

Given that EGFR is expressed on normal cells, it would be worthwhile to target variants of EGFR that are only expressed on cancer cells. One such variant is EGFR variant III (EGFRvIII) where exons 2–7 are deleted from wt EGFR [18]. This in-frame deletion produces a truncated extracellular domain and introduces a novel glycine at the new junction [18] EGFRvIII exhibits persistent low-level tyrosine kinase activity. Of interest, EGFRvIII is expressed only in tumor tissue and detected in ~30% of biopsies from glioblastoma multiforme (GBM) patients. The median survival of GBM patients is around 2 years with the current standard of care [19]. Given this poor prognosis, there is an urgent need for new treatments and EGFRvIII constitutes an attractive target [20].

In terms of targeting EGFRvIII-expressing cells with immunotoxins, two have been evaluated in clinical trials. A murine based scFv, MR1, was isolated against a peptide that included the novel glycine at the exon 1-8 splice junction of EGFRvIII. The scfv was fused with PE38 to make MR1(Fv)-PE38. MR1(Fv)-PE38 killed glioblastoma cells both in vivo and in vitro [21]. The binding affinity of MR1 for EGFRvIII was improved through targeted mutagenesis of CDR3 sequences resulting in MR1-1 which had a 15-fold higher binding affinity and a 3.5-fold increase in potency [21,22]. The improved immunotoxin entered a Phase I trial which was terminated due to low patient accrual (ClinicalTrials.gov Identifier: NCT01009866).

In GBM, poor responses can be due to molecular heterogeneity where the tumor often includes cells with either EGFRvIII or wt EGFR expression [23]. Therefore, a therapeutic agent that only targets EGFRvIII might not reduce tumor burden in a substantial way [24,25]. Immunotoxins with dual specificity for both EGFRvIII and EGFR might overcome this limitation. The D2C7 antibody was shown to react with both EGFRvIII and wild type EGFR [26]. A disulfide-stabilized scFv of D2C7 fused with PE38 was further modified with the addition of a “KDEL” sequence at the carboxyl terminus to generate, D2C7(scdsFv)-PE38KDEL or D2C7-IT for short [27]. The addition of the KDEL sequence was to allow more efficient retrograde transport of the toxin to the endoplasmic reticulum. D2C7-IT displayed a high level of killing against tumor cell lines expressing EGFR, EGFRvIII or a mixture of both EGFR/EGFRvIII [27]. In vivo D2C7-IT reduced tumor growth where survival was enhanced by 166% versus control [27]. Following a successful GLP study of D2C7-IT in rats, D2C7-IT entered a Phase I/II clinical trial for patients with recurrent malignant glioma (ClinicalTrials.gov Identifier: NCT02303678) [28,29]. Another clinical trial using D2C7-IT, currently in Phase I, examines the efficacy of combining D2C7-IT with checkpoint inhibitor Atezolizumab in recurrent GBM patients (ClinicalTrials.gov Identifier: NCT04160494). These trials are ongoing.

Given the heterogeneity of GBM gene expression and the potential value of dual targeting antibodies against EGFR and EGFRvIII, additional efforts have been made to make immunotoxins that include this type of reactivity. The 806 monoclonal antibody, mab806, originally raised in mice, was shown to bind to EGFRvIII as well as EGFR when overexpressed [30,31]. Antibody reactivity was directed to a conformational-dependent loop (aa 287–302) in the extracellular domain that is fully exposed in EGFRvIII and partially exposed in cells overexpressing EGFR [32]. Further, this loop is masked in active or dimerized EGFR [32]. This allowed for mab806, and antibodies derived from it, to target not only EGFRvIII but also overexpressed EGFR when expressed on particular cancerous cells [32]. The utility of this antibody was examined by constructing an immunotoxin and testing its activity against triple-negative breast cancer with EGFR amplification/overexpression but no expression of EGFRvIII. Regarding triple-negative breast cancer, EGFR amplification has been reported to occur in approximately 60% of cases when analyzed by silver in situ hybridization, validating this receptor as a target in this malignancy [6]. The scFv of 806 was fused with PE38 to produce the 806-PE38 immunotoxin [33]. In vitro analysis indicated that it killed triple-negative breast carcinoma cell lines with EGFR overexpression but not lines that only expressed wildtype EGFR at normal levels [33]. In vivo data with xenografts showed tumor size reduction and increased survival when mice were treated with 806-PE38 [33].

More recently, efforts were made to produce another dual specificity antibody with improved properties that targets the same EGFR conformational loop (aa 287–302). Given the identity of the cryptic epitope, a more targeted approach was made by producing antibodies against KLH-conjugated peptide corresponding to the (aa 287–302) loop [34]. A new antibody, 40H3, was raised and in vitro data suggest that it binds to both EGFRvIII and overexpressed EGFR but not wild type EGFR [34]. Its immunotoxin, 40H3-PE38, was also shown to kill both EGFRvIII, and EGFR overexpressing cell lines [34].

## 5. HER2

Efforts have also been made to generate immunotoxins against another member of the ErbB family, ErbB-2 or HER2. Although structurally similar to other members of the ErbB family, no binding ligand has been identified for HER2. Without a known ligand, the development of targeted toxins against HER2 has been antibody-based. One of the first anti-HER2 immunotoxins, Erb-38, was made by fusing the dsFv of anti-HER2 antibody, Mab e23, with PE38 [35,36]. Erb-38 killed HER2 expressing tumor cell lines and caused complete regressions in nude mice bearing epidermoid or breast carcinomas [36]. A Phase I trial was initiated in patients with advanced breast (five patients) or esophageal cancer (one patient) all of whom had failed standard therapy [35]. However, dose-limiting hepatotoxicity was observed in all patients due to unappreciated HER2 expression on hepatocytes [35]. Given the resulting liver toxicity, the clinical trial was terminated.

Another HER2 directed immunotoxin that reached clinical trial was scFv(FRP5)-ETA, also known as Zemab [37,38]. In vitro data indicated that Zemab displayed potent cytotoxicity against both established and primary human tumor cells including breast, ovarian, and squamous cell carcinomas [38,39]. In vivo data indicated that Zemab successfully inhibited the growth of human tumor xenografts in mice when the immunotoxin was applied both locally and systemically [38,40,41]. Zemab was evaluated in a Phase I trial of patients (*n* = 18) with metastatic breast, prostate, head and neck, non-small cell lung, or transitional cell carcinoma [42]. Although dose-limiting hepatotoxicity was observed in three of the patients, two patients showed stable disease while three saw responses (such as lesion healing and lymph node size reduction) [42]. Of note, only five of eleven patients produced neutralizing antibodies to the immunotoxin [42]. For strategic reasons, no further development of Zemab was pursued (Citeline Informa: Zemab). HER2 has also been targeted with the “herceptin-based” scFv designated 4D5 fused to recombinant gelonin, with cytotoxic activity achieved in both tissue culture and tumor xenografts [4].

Currently, there is one anti-HER2 immunotoxin, MT-5111, that is being evaluated in a Phase I trial for patients with HER2-positive breast cancers (ClinicalTrials.gov Identifier: NCT04029922). MT-5111 is composed of an scFv from the anti-HER2 antibody that binds to a different region from either pertuzumab or trastuzumab [43]. It was fused with a de-immunized Shiga-like toxin-A subunit. This immunotoxin was cytotoxic for cell lines expressing HER2 but resistant to trastuzumab (i.e., HCC1954) or T-DM1 (i.e., JIMT-1 and gastric SNU-216) [43,44]. A Phase I clinical trial is currently underway but no data are available yet.

## 6. HER3

Unlike EGFR and HER2, HER3 has not been widely targeted by immunotoxin-type agents. Recently, however, Capone et al. reported a saporin conjugate with high potency for HER3-expressing melanomas. The EV-20 antibody, conjugated to saporin via a disulfide bridge, exhibited an IC50 in viability assays of 0.2 nM when added to SK-MEL2 cells [45].

## 7. UPAR and Bispecific Targeting of EGFR

Because of potential toxicity to normal tissue, ligand-toxin proteins have been modified. One modification was the addition of the amino-terminal fragment (ATF) of urokinase with the goal of bispecific targeting to cells with dual expression of EGFR and urokinase plasminogen activator receptor (uPAR) [46]. The recombinant ligand toxin termed eBAT has shown promise as a targeting agent for tumors with an expression of both EGFR and uPAR [47,48]. Small and large animal data suggest eBAT has fewer toxicity problems than EGF-toxin [49,50,51]. The clinical trial evaluation will be needed to determine utility for this agent against a variety of solid tumors.

Targeting of uPAR is also possible with a single binding specificity. ATF-saporin was constructed as a gene fusion and expressed in *Pichia pastoris*. When added to uPAR expressing leukemic cells, this ligand toxin proved to be a potent cell-killing agent [52]. More recent studies have expanded to include more tumor types, including bladder cancer [53].

## 8. Immunotoxins Targeting the Transferrin Receptor

Receptors for nutrients including lipid or protease metabolites are known to be internalized efficiently. When these receptors are present at high levels on cancer cells, they constitute valid targets. The transferrin receptor has been studied intensively both as a model and as a clinical target [54]. Transferrin is the main protein that regulates and distributes circulating iron. Further, the transferrin receptor is overexpressed in many cancers where increased expression is correlated with a poor prognosis [55]. High receptor expression may be due to an increased iron requirement [56] leading to a high turnover of the transferrin receptor [57]. Therefore, transferrin-mediated endocytosis and the subsequent recycling pathway are highly efficient in cancer cells [58,59,60,61].

Both plant and bacterial toxins have been chemically conjugated or fused through DNA cloning to transferrin or to anti-transferrin receptor antibodies to deliver toxic payloads to cancer cells. The ribosome-inactivating proteins are potent plant toxins. Through their *N*-glycosidase activity, they remove specific purine bases from the sugar moiety of the 28S ribosome, arresting protein synthesis at the translocation step [60,61,62]. Ricin, gelonin and saporin have been conjugated to both transferrin and monoclonal antibodies against the human transferrin receptor and tested in different cancer settings. Anti-transferrin receptor antibody-ricin and transferrin-ricin immunotoxins have been tested in a variety of cancer settings where their cytotoxic activity was demonstrated to be highly potent, tumor cell type-specific and correlated with the expression of the transferrin receptor [63,64]. In vivo studies showed that ricin immunotoxins reduced tumor growth in xenografts model of human glioblastoma and melanoma [63,65,66]. The effectiveness of the ricin immunotoxin, 454A12-rRA, in murine models of glioblastoma was the rationale for a Phase I human clinical trial in eight patients with leptomeningeal spread of systemic malignancies. Four out of the eight patients responded to treatment, with a 50% or greater reduction of tumor cell counts in their lumbar cerebrospinal fluid; however, no patient had their CSF cleared of tumors, and tumor progression was demonstrated in seven of the eight patients after treatment [65]. Saporin transferrin-targeted immunotoxins were able to enter into the cells via TfR binding and inhibit cell proliferation of leukemia, HepG2, GL-15 and U87 glioblastoma multiform human cell lines [67,68,69]. An immunoconjugate containing the monoclonal antibody (5E9), against the human transferrin receptor and the protein gelonin was extremely toxic to human leukemia, lymphoma and cervical cancer cell lines. In a xenograft model of Burkitt’s lymphoma, it prolonged survival and delayed or prevented the growth of subcutaneous nodules [70].

Chemically coupling Pseudomonas exotoxin A to anti-transferrin receptor antibodies or fusing them recombinantly generated potent immunotoxins that inhibited protein synthesis and induced cell death in human ovarian and breast carcinoma cell lines and primary cells from ovarian metastasis [71,72,73]. The efficacy of PE-based immunotoxins was also confirmed in vivo in a nude mouse model of human ovarian cancer [74], A431 epidermoid tumors [75] and colon cancer [76]. While no clinical trials followed the preclinical results, the anti-transferrin receptor-PE immunotoxin has been an excellent tool to elucidate the mechanism of resistance to PE-based immunotoxins. For instance, it was shown that protein synthesis inhibition by immunotoxins decreased not only the well-known prosurvival Bcl-2 family member, Mcl-1, but also the level of three major proapoptotic BH3-only proteins, Bim, PUMA and Noxa but only Bim protein levels correlated with the ability of immunotoxins to induce an apoptotic response [77].

In another study, a point-mutant form of diphtheria toxin (DT), CRM 107, which reduces the affinity for the DT receptor compared to native toxin [78], was conjugated to transferrin [79]. This transferrin-DT conjugate, Tf-CRM107, was evaluated in patients with malignant brain tumors. In Phase I testing, the protein was delivered directly into the tumoral mass with high-flow interstitial microinfusion, bypassing the blood–brain barrier, obtaining high local drug concentration in the tumor, and reducing systemic toxicity. At least a 50% reduction in tumor volume, determined by magnetic resonance imaging occurred in 9 of 15 patients who could be evaluated (60%), including two patients with a complete response. One patient remained tumor-free for 24 months after treatment. Peritumoral toxicity developed 1-4 weeks after treatment only in patients treated with the highest dose of the immunotoxin (1.0 ug/mL), but no toxicity was detected in patients treated at lower concentrations [80]. The Phase II study was a multicenter trial using intratumoral infusions of Tf-CRM107 to treat refractory and recurrent glioblastoma and anaplastic astrocytoma [81]. The results of the Phase II clinical trial confirmed the safety and tumor response data of the Phase I trial: Tf-CRM107 treatments resulted in both complete and partial tumor responses without severe toxicity in 35% of the evaluable patients. Two Phase III clinical trials were initiated: one was completed with no results posted (ClinicalTrials.gov Identifier: NCT00088400) and the other one (ClinicalTrials.gov Identifier: NCT00083447) was withdrawn prior to recruitment of patients because Tf-CRM107 would not meet trial criteria for efficacy. A Phase I trial where children who have progressive or recurrent glioblastoma multiforme or anaplastic astrocytoma, were treated with Tf-CRM107 was opened but the status is unknown (ClinicalTrials.gov Identifier: NCT00052624).

## 9. Immunotoxins Targeting Cytokine Receptors

Cytokine receptors along with their ligands contribute to cancer progression and can be targeted by ligands or anti-receptor antibodies. While two of the most prominent include the IL2 and IL3 receptors (there are approved agents targeting each of these receptors), others that clearly warrant study include IL4R, IL6R, IL7R and IL13R. For this review, the IL2 and IL3 receptors will be considered first and then the remainder of the cytokine receptor family. Both DT and PE cytokine-toxins have been produced as gene fusions but similar constructs have not been described for the plant toxins due to constraints posed by constructing gene fusions that retain both ligand binding and toxin activity. Of clinical interest, two of the DT fusions have been approved for human use. The approval of DT-IL2 (DAB389-IL2) was granted for the treatment of cutaneous T-cell lymphoma and DT-IL3 (DAB389-IL3) for the treatment of blastic plasmacytoid dendritic cell neoplasm.

## 10. Targeting the Interleukin-2 Receptor (IL2R)

Interleukin-2 was one of the first cytokines described for T-cell activation [82]. In the years that followed its discovery, the biology of its receptor, a heterotrimer, was gradually uncovered. We now know that the IL2R comprises an alpha chain (identified via its interaction with the anti-Tac antibody and now also called CD25), a beta chain (CD122) and a gamma chain (CD125). The high-affinity binding of IL2 requires all three chains. For targeting IL2R, early ligand-toxin constructs included DT-IL2, IL2-PE40 and immunotoxins constructed with the anti-Tac antibody joined with various PE fragments [83,84,85,86]. Aberrant IL2R expression occurs in several malignant hematopoietic malignancies including adult T-cell leukemia and hairy cell leukemia and thus this receptor is a legitimate cancer target. However, CD25 is also expressed on (non-malignant) regulatory T-cells and thereby constitutes a target, while relevant to cancer therapy, that is in fact expressed on normal cells. DT-IL2 was produced first from a longer DT transcript and was later shortened to DT389-IL2. Preclinical studies and various clinical trials led to its approval as Denileukin diftitox for the treatment of cutaneous T-cell lymphoma [87,88]. Despite its early promise, Denileukin diftitox has been discontinued in the US.

Ricin-based immunotoxins were also produced targeting IL2R. A second-generation immunotoxin, RFT5-SMPT-dgA, which joined the whole anti-CD25 monoclonal antibody RFT5 to deglycosylated ricin A-chain, was evaluated in patients with refractory Hodgkin’s lymphoma, with some partial remissions [89,90]. A later study showed that RFT5-SMPT-dgA induced a decrease in Treg cells in six metastatic melanoma patients [91]. To date, ricin-based immunotoxins targeting IL2R have not been approved for human use.

With the advent of scFv fusions, the anti-Tac immunotoxin was recreated as LMB-2 and has been developed both for treating leukemias and eliminating T-reg cells [92,93]. The latter treatment strategy is an approach to reduce immune suppression and allow effective T-cell responses to tumor antigens. In cancer settings, LMB-2 has produced objective responses in both adult T-cell leukemia and hairy cell leukemia, but regulatory approval has not yet been achieved.

## 11. Targeting Interleukin-3 Receptor (IL3R)

The human interleukin-3 receptor (IL3R) is a cell-surface heterodimer composed of α-subunit domains and an N-terminal domain with unknown function. The α subunit is essential for ligand binding and confers specificity on the receptor. IL3R also has a common γ (γc) subunit. The γc subunit is shared by the granulocyte macrophage-colony stimulating factor (GM-CSF) and IL-5 receptors and is required for high-affinity ligand binding and signal transduction. IL3 is a pleiotropic cytokine produced mainly by activated T lymphocytes and physiologically stimulates the production and function of multiple hematopoietic cells including dendritic cells. The IL3R is overexpressed in acute (AML) and chronic myeloid leukemia (CML) progenitor cells. IL3R is also detected on subpopulations of AML blasts enriched for malignant progenitors that engraft nonobese diabetic severe combined immunodeficient (NOD/SCID) mice [94,95]. The immunotoxin targeting IL3R is constructed via fusing human IL3 to a truncated form of diphtheria toxin (DT388) [96]. DT388-IL3 showed antitumor activities in an SCID model of acute myeloid leukemia (AML). More recently, this molecule was developed for Phase I trials in patients with chemorefractory AML and myelodysplasia (MDS) [97]. A commercially available construct composed of human IL3 and a truncated diphtheria toxin was developed by Stemline Therapeutics, Inc. the compound, called Tagraxofusp (tagraxofusp-erzs-Elzonris™), is used for the treatment of blastic plasmacytoid dendritic cell neoplasm. It was approved in 2008 in the USA for the treatment of adults and pediatric patients aged 2 years and older [98].

## 12. Targeting the Interleukin-4 (IL4R) and Interleukin-13 (IL13R) Receptors

The interleukin-4 receptor (IL4R) is a type I cytokine receptor. It is composed of the IL4R α chain and the common γ chain (γc). The binding of ligand activates a signaling cascade that leads to phosphorylation events mediated by receptor-associated kinases and, in turn, these recruit mediators of cell growth, resistance to apoptosis and gene activation [99]. Two ligands that bind this receptor are IL4 and IL13. IL4 and IL13 are pleiotropic cytokines produced by a variety of cell types. They are known as Th2 cytokines that mediate host defense against parasites, allergies and inflammatory responses. IL4Rs are expressed on the surface of several human cancers including melanoma, ovarian and breast carcinoma [100], neurological cancers [101], head and neck cancers [102], bladder cancer [103], renal cell carcinomas [99] and AIDS-associated Kaposi’s sarcoma cells [104]. In contrast, human B cells, monocytes, T cells, and endothelial cells express undetectable or low amounts of these receptors [105]. IL4R is composed of several distinct chains [106], on several tumor cells IL4Rα and IL13Rα’ chains are expressed and bind IL4, while in many solid tumor IL13 binds the chains IL-4Rα and IL13Rα1 and IL13Rα2 [107]. The functional overexpression of IL4Rα and its ligand on several human cancer cells has been the rationale for generating immunotoxins targeting IL4R and IL13R [108]. These immunotoxins are composed of an IL4 or IL13 moiety and a truncated fragment of PE. IL4-PE was developed as a circularly permuted protein: the single-chain circularly permuted IL-4 mutant IL4(38-37) was created, containing aa 38-129 of IL-4, a GGNGG linker and aa 1-37 of IL-4 which are then fused to the toxin [109]. This immunotoxin was highly and specifically toxic to tumor cells, including brain tumor cells, and showed in vivo antitumor activity in animal xenograft models of a variety of human cancers [107,108,109]. The clinical efficacy of IL4-PE was reported for the first time by Rainov and Heidecke and Rand, et al. [110,111] in the treatment of patients with recurrent malignant glioma following intracranial infusion of cytotoxin [109]. Moreover, this immunotoxin showed strong cytotoxic activity against IL-4R-positive carcinomas in pre-clinical studies and several studies have been conducted in patients with several types of solid tumors. IL-4-PE in combination with gemcitabine was targeted to pancreatic carcinoma cells [107,112,113]. This immunotoxin showed a potent in vitro and in vivo antitumor activity against human biliary tract carcinoma.

In addition to these PE fusions, a DT-IL4 fusion protein was produced from the murine IL4 gene (DT389mIL4) and was reported as an immunosuppressive agent that blunted delay-type hypersensitivity responses in mice [114].

Concerning IL-13, it was incorporated into another immunotoxin targeting IL-4/13R, called IL-13-PE38QQR (also known as cintredekin besudotox or IL13-PE). IL-13R, as mentioned above, consists of an IL-13Rα1 chain and IL-4Rα and the signaling is similar to IL-4R. Physiologically, IL-13 receptors are expressed on human B cells, basophils, eosinophils, mast cells, endothelial cells, fibroblasts, monocytes, macrophages, respiratory epithelial cells, and smooth muscle cells [115]. To generate IL-13 cytotoxin, the NH_2-_terminal domain, especially the Glu^13^, (binding ubiquitously to receptors on eukaryotic cells) was removed to avoid specific binding, and the DNA sequence encoding IL-13 was inserted upstream of PE. Three amino acids (K590, K606, and K613) in domain III were replaced by Q, Q, and R, respectively. IL13-PE was highly selective and potent for killing human tumor cells in vitro, in particular glioblastoma cells that expressed high levels of IL-13Rα2 chain. Since this chain is undetectable in immune cells, endothelial cells, and normal human astrocyte, the killing of tumor cells is more specific [113]. Most studies were conducted in vitro on glioma cell lines and glioma cells derived from patients and xenografts models [116]. This immunotoxin was evaluated in various trials from Phase I to III. In most of these trials, IL13-PE was administered via convection-enhanced delivery, a method of direct drug delivery, optimizing the distribution of macromolecules within the brain [116,117,118,119]. In 2004, another immunotoxin was generated whereby a mutant of human IL-13, in which a glutamic acid (E) residue at position 13 was substituted by a lysine (K) residue (this mutant was termed IL-13E13K and was fused to a mutated form of PE). This fusion protein had an increased binding to IL-13Rα2 and showed more cytotoxicity at lower doses compared to wild type, in studies conducted on glioma cell lines in vitro and in xenografts models [120]. IL-13-PE38QQR was cytotoxic for human renal cell carcinoma cells [121], prostate cancer cells [122] and Squamous Cell Carcinoma of Head and Neck tumor cell lines. Based on the selectivity of IL-13α2 expression, another immunotoxin was developed, DT-IL13QM. In this construct, a quadruple-mutated IL-13-based construct was fused to the first 389 amino acids of DT. This ligand toxin was more specifically cytotoxic to glioblastoma multiforme cells and less toxic in general, in an intracranial rodent model compared to a flank tumor model in previous studies [123,124].

A bispecific immunotoxin targeting IL-13 and uPAR, called DTAT13, was also constructed. This immunotoxin can bind IL-13R expressing cells and uPA-expressing tumor neovasculature. In vivo, DTAT13 caused the regression of small tumors and was able to target both overexpressed uPAR and the vasculature, as demonstrated by activity against HUVEC cells [125].

Another bispecific immunotoxin targeting IL-13R and EGFR (DTEGF13) was constructed in order to react with overexpressed receptors on cancer cells and on normal cells. This cytotoxin was generated to reduce binding to normal receptors while still targeting receptors over-expressed on cancer cells thereby decreasing toxicity while maintaining efficacy, The DTEGF13 promotes efficacy in a model of orthotopic pancreatic cancer, showing promise for treating refractory pancreatic carcinomas [126]. This cytotoxin also has activity against other tumors, e.g., in xenografts models of prostate cancer [127] and by intracranial injection in a glioblastoma rat model [50].

## 13. Targeting Interleukin-7 Receptor (IL-7R)

The IL7 receptor (IL7R) is formed from IL7Rα (CD127) and the common cytokine receptor gamma chain (γ-chain, CD132). The binding of IL7 induces proliferative and anti-apoptotic signals mainly by activating the JAK-STAT pathway [128]. IL-7R is mainly expressed on conventional mature T lymphocytes (except on T-regs, where expression is low) while IL-7 is mainly produced by epithelial and stromal cells. The deregulation of IL7/IL7R signaling can promote cancer development [129]. In particular, this deregulation is associated with leukemogenesis, e.g., acute lymphoblastic leukemia (ALL) of T- and B-cell origin development [130,131] and chronic lymphocytic leukemia [132]. To target lymphoblastic cancer cells, DAB389IL7, was constructed. DAB389IL7 immunotoxin targets malignant cells expressing the IL-7R, such as cutaneous T lymphoma, acute T cell leukemia, acute and chronic B cell malignancies, Burkitt’s lymphoma, and Hodgkin’s disease.

## 14. Targeting Interleukin-6 Receptor (IL6R)

The interleukin-6 receptor (IL6R or CD126) recognizes and binds interleukin-6 (IL6), a multifunctional cytokine with both pro- and anti-inflammatory properties. The IL6 signaling cascade is initiated by binding of IL6 to IL6R and a second transmembrane protein, gp130 (ubiquitously expressed) [133]. Under non-pathogenic conditions, IL6R expression is restricted to hepatocytes, monocytes, and lymphocytes. The IL6/IL6R interaction activates JAK-mediated phosphorylation of STAT3 and the formation of STAT3 homodimers. Additionally, IL6 activates MAP-kinase and the phosphatidylinositol-3 kinase (PI3K)/protein kinase B (AKT) pathways [134]. Excessive IL-6 production and dysregulation of the IL6/IL6R axis can lead to inflammation or cancer. During cancer progression, high levels of both IL6 and ILR6 have been reported [135,136]. IL6 signaling plays an important role in tumor cell growth, survival, angiogenesis, immunomodulation of the tumor microenvironment, stromal cell activation, and cancer progression [137]. In the tumor microenvironment, IL6/JAK/STAT3 signaling drives proliferation, survival, invasiveness, and metastasis of tumor cells, while strongly suppressing the anti-tumor immune response [138]. IL6 and IL6R also play important roles in the development of several hematological malignancies, including myeloma, B-cell leukemias, lymphomas and non-B cell malignancies.

To target IL6R-expressing cells a recombinant fusion protein, IL6-PE^4E^, was constructed from human IL6 fused to a mutated PE. The mutations in PE prevented binding to the PE receptor. This chimeric toxin was potently cytotoxicity against a variety of different tumor cell lines displaying IL6Rs including multiple myeloma, prostate, hepatoma and carcinoma [139]. In two studies of pediatric cancer, IL6-PE was cytotoxic for freshly isolated (IL6R-expressing) patient cells diagnosed with either Acute Myelogenous Leukemia or Acute Lymphoblastic Leukemia [140,141]. The killing was roughly proportional to the number of receptors per cell.

To target multiple myeloma, another IL6-based immunotoxin was constructed, called IL6(T23)-PE38KDEL, with greater potency than IL6-PE40 in vitro and fewer side effects than IL6-PE^4E^ [142]. This immunotoxin is a chimeric molecule composed of interleukin 6 (IL6), missing the N-terminal 23 amino acids, and fused to a truncated mutant form of *PE* (PE38KDEL). The antitumor activity of IL6(T23)-PE38KDEL depended not only on IL6R, but also on the levels of IL6 and soluble IL6R.

## 15. Other Receptors

### 15.1. Targeting C-C Chemokine Receptor Type 9 (CCR9)

CCR9 belongs to the β-chemokine receptor family and is expressed on immature T lymphocytes, regulating their development and tissue-specific homing and on intestinal cells [143]. The ligand for this receptor is the chemokine CCL25, mainly expressed in thymus and by epithelial cells of the small intestine. CCR9 expression is increased in various cancers (breast, prostate, ovarian and lung cancer [144]), and CCL25/CCR9 signaling has been found in several tumors. Moreover, the expression of CCR9 and has been associated with tumor chemoresistance and metastasis [145]. CCL25 fused to PE38 was constructed to target CCR9-bearing cancer cells including CCR9-high-expressing human T-ALL cells. However, this immunotoxin did not eliminate all cancer cells, as some parts of the expanding tumor did not express CCR9, rendering CCL25-PE38 less than completely effective [145].

### 15.2. The EPH Receptors

Some of the ephrin receptor family of protein–tyrosine kinases (there are 14 known receptors) are expressed at high levels in cancers, leading to the design of several immunotoxins. Ephrin B2 receptor expression was targeted on colorectal cancer cells with an antibody–drug conjugate [146], gliomas were targeted with an Ephrin A2 receptor immunotoxin [147]. Ephrin A3 receptor and ephrin A2 receptors were also targeted [148,149].

### 15.3. MSH Receptors

The Murphy Lab began the gene fusion approach for targeting toxins to receptors by fusing portions of melanocyte-stimulating hormone (MSH) with truncated DT [150]. The goal was to produce fusion proteins that would kill malignant melanoma cells expressing the alpha-MSH receptors [151]. DT-MSH was not evaluated in clinical trials. However, a similarly conceived molecule was reported more recently as a fusion protein with truncated PE (MSH-PE38KDEL) [152]. It is not clear if this molecule will be developed as a clinical candidate.

## 16. Conclusions

Receptors that are expressed at high levels on cancer cells are attractive targets for ligands or antibodies that are armed with toxic payloads. Potent payload types include enzymatically active protein toxins such as plant or bacterial toxins. Here, we summarized several decades of research and development where receptors that internalize efficiently were targeted with chimeric therapeutic molecules. At least two of these ligand-toxin molecules, DAB389IL2 and DAB389IL3, have been approved with several other candidates in late-stage clinical evaluation. Recently, a PE-based immunotoxin, targeting CD22, was approved for treating patients with hairy cell leukemia [153,154]. Because many of these patients were immunosuppressed, multiple cycles of therapy could be given before anti-toxin antibodies were produced. This has not been the case when treating patients with solid tumors, where anti-toxin antibodies frequently appear after one or two cycles with immunotoxin agents. Going forward, two issues must be addressed: the immunogenicity of the toxin portion of the molecule and toxin-mediated damage to normal tissue expressing target receptors. The former is being addressed by using mutant toxins with fewer immunogenic epitopes [155]. Damage to normal tissues can be addressed with improved antibody specificity or with the use of immunotoxin-enhancing agents that, in combination, target malignant cells over normal cells. Screening efforts for immunotoxin enhancers are underway [156].

## Abbreviations

DT	diphtheria toxin
PE	pseudomonas exotoxin
EGFR	epidermal growth factor receptor
EGFRvIII	EGFR with a deletion of exons 2–7
DT388/DAB388	truncated DT including the first 388 amino acids
PE40	truncated PE including domains 2 and 3
PE38	truncated PE including domains 2 and 3 without a disulfide loop
TP40	Transforming growth factor alpha-PE40
scFv	antibody, single-chain Fv
ETA	exotoxin A (alternative name for PE)
ATF	amino-terminal fragment
uPAR	urokinase plasminogen activator receptor
LMB-2	immunotoxin from the scFv of the antiTac antibody and PE40
